# Whey Protein Dietary Supplements: Metal Exposure Assessment and Risk Characterization

**DOI:** 10.3390/nu15163543

**Published:** 2023-08-11

**Authors:** Elena Bethencourt-Barbuzano, Dailos González-Weller, Soraya Paz-Montelongo, Ángel J. Gutiérrez-Fernández, Arturo Hardisson, Conrado Carrascosa, Montaña Cámara, Carmen Rubio-Armendáriz

**Affiliations:** 1 Interuniversity Group of Environmental Toxicology, Food and Drug Safety, University of La Laguna, 38071 La Laguna, Spain; alu0101115948@ull.edu.es (E.B.-B.); spazmont@ull.edu.es (S.P.-M.); ajguti@ull.edu.es (Á.J.G.-F.); atorre@ull.edu.es (A.H.); conrado.carrascosa@ulpgc.es (C.C.); 2 Health Inspection and Laboratory Service, Canary Health Service, 38006 Santa Cruz de Tenerife, Spain; dgonzal@ull.edu.es; 3 Department of Animal Pathology and Production, Bromatology and Food Technology, Faculty of Veterinary, University of Las Palmas de Gran Canaria, 35413 Arucas, Spain; 4 Nutrition and Food Science Department, Complutense University of Madrid, 28040 Madrid, Spain

**Keywords:** protein supplements, essential minerals, potentially toxic elements, contaminants in food

## Abstract

Protein supplements (PS) are trendy foods, especially those made from whey. In addition to providing protein, these products are a source of metals, providing essential elements (Na, K, Mg, Ca, Mo, Mn, Fe, Co, Cu, and Zn) and other potentially toxic elements (Al, B, Sr, V Ba, and Ni). In this study, 47 whey PS samples were analyzed for mineral elements by ICP-OES, and their dietary exposures were assessed for three consumption scenarios. Elements found in higher concentrations were K (4689.10 mg/kg) and Ca (3811.27 mg/kg). The intake of 30 g PS (average recommended amount/day) provides about 20% of the established reference value (NRI) for Cr (18.30% for men and 25.63% for women) and Mo (26.99%). In a high daily consumption scenario (100 g PS/day) and when the maximum concentrations are considered, Cr, Zn, Fe, Mo, and Mg dietary intakes of these metals exceed the daily recommended intakes and could pose a risk. The daily intake of 30, 60, and 100 g of whey PS for 25 years does not pose a health risk since the hazard index (HI) is less than one in these consumption scenarios, and the essential elements contributing most to HI are Co, followed by Mo and Cr. It is recommended to improve the information to the consumers of these new products. Furthermore, to help in the management and prevention of these potential health risks, it would be advisable to improve the regulation of these dietary supplements and their labeling.

## 1. Introduction

Athletes currently find a wide variety of dietary supplements on the market. During the last decades, the sector of these sports supplements has increased considerably. Among the reasons is the emergence of new and diverse consumer profiles attracted by new dietary trends. Some consumers even believe that these supplements are useful to make up for nutritional deficits resulting from incorrect dietary habits [1,2,3]. Dietary supplements are known for providing different nutrients to supplement the diet, and they are usually consumed with the aim of increasing muscle mass, improving physical performance, replenishing nutrients or energy, and maintaining an adequate state of health [1,4,5,6].

Among the different types of nutritional supplementation available, the most widely used by athletes are protein supplements (PS), especially those formulated from whey, as they have proven to increase the rate of muscle synthesis after training more effectively [7,8,9].

The global protein supplements (PS) market was estimated to be around 20.19 billion USD in 2021, but it is expected to exceed 49.29 billion USD by 2030 [10]. In Europe, dietary supplements market figures are estimated at 31 billion USD by 2027 [11]. The COVID-19 pandemic may have reinforced this trend since the population associated the consumption of PS to strengthen their immune system [10,11]. The benefits associated with the consumption of PS are relatively known, and, in general, consumers consider them safe. However, there is growing concern about their use, especially under chronic high consumption, because the population finds a wide range of these products on the market that can be used without being subject to the supervision of a health professional such as a pharmacist or nutritionist [12,13].

Different types of PS are available on the market; isolates have a high protein content with a lower amount of carbohydrates and fat [14,15]. The main component of PS is protein, but essential mineral elements as sodium (Na), potassium (K), magnesium (Mg), calcium (Ca), molybdenum (Mo), manganese (Mn), copper (Cu), cobalt (Co), zinc (Zn), and chromium (Cr); and potentially toxic elements (PTE) (aluminum (Al), boron (B), strontium (Sr), vanadium (V), barium (Ba), and nickel (Ni)) have also been detected although there are very few studies evaluate their occurrence [7,16,17,18].

Within the essential elements, the most abundant cation in the extracellular body fluid is Na and is involved in the maintenance of cellular homeostasis and excitability, although its excess is associated with cardiovascular pathologies [19,20,21]. K compensates for the effects of Na on blood pressure as well as being involved in the proper functioning of the nervous and cardiovascular systems and muscles [22,23].

Mg maintains the hydroelectrolyte balance and acts as an enzymatic cofactor, as Ca, and its deficiency is associated with neurological, muscular, and renal alterations and excess gastrointestinal alterations [24,25,26]. Ca plays a key role in the coagulation cascade, and it is closely related to boss mass [25,26].

Among the essential microelements, Fe transports oxygen in the body as part of hemoglobin, but excessive exposure causes oxidative damage [27,28]. Cu is part of different enzymes involved in Fe metabolism, leading to anemia in situations of deficiency and liver disease in excess [28,29]. Cr and Ni are considered a Group 1 carcinogen by the International Agency for Research on Cancer (IARC) but also have been associated with gastrointestinal alterations due to their irritant effect and hepatic alterations [30,31,32,33,34,35].

Mo is essential for the processing of proteins and DNA in the body; a deficit of this metal causes neurological and growth disorders [36,37,38]. Mn is considered an essential component as a cofactor for numerous metalloenzymes involved in metabolism, and it is related to the nervous system [39,40]. Co is essential because it is a component of vitamin B12, which is involved in multiple biological functions [41]. Co-poisoning is associated with weight loss, loss of appetite, weakness, increased hemoglobin, and red blood cell counts [42]. Zn is involved in the growth and proper maturation of the associated immune system [43]. However, high intakes are associated with dizziness, headaches, vomiting and loss of appetite [44,45].

Some elements are potentially toxic as Sr has a high affinity for Ca, being useful for osteoporosis but, in excess, causes kidney damage [46,47,48]. High doses of Ba cause cardiac and renal problems, alterations in blood pressure, paralysis, and muscle weakness [48].

Al is a metal that has been associated with neurotoxicity when the patient is exposed to it parenterally and other disorders [49,50,51].

Finally, B, once absorbed, accumulates in the bone, and its excess cause reproductive disorders [52]. V is not essential, but studies have shown that it increases insulin sensitivity, but its deficiency and excess cause bone and gastrointestinal disorders [53,54].

For all this, the main goal of this work is to perform a metal-exposure assessment and risk characterization of whey protein dietary supplements in order to establish their safety in different consumption scenarios considering the Nutritional Reference Intakes (NRIs) established for the Spanish population for essential elements (Table 1) [55,56]. Likewise, Table 2 presents for potentially toxic elements (PTE), the intakes set by the European Food Safety Authority (EFSA), together with other authorities, that should not be exceeded (Tolerable Daily Intake (TDI), Tolerable Weekly Intake (TWI), Tolerable Upper Intake Level (UL)).

## 2. Materials and Methods

### 2.1. Samples

A total of 47 whey PS samples were analyzed, with protein percentages declared on the label ranging from 18 to 93%. These samples were purchased between March 2021 and April 2022, mainly in Spain and Italy.

**Figure 1:**
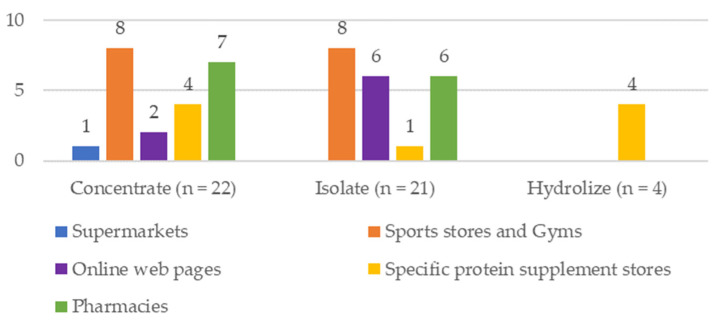
Description of the analyzed samples of whey Protein Supplements.

According to the type of protein, 46.80% of the PS was concentrated, 44.68% isolated, and 8.51% hydrolyzed. The PS were mostly of European origin and were collected in different establishments (supermarkets (2.13%), sports stores and gyms (34.04%), online web pages (17.02%), and specific protein supplement stores (19.15%)) (Figure 1). A specific store is defined as a store that sells only one or two products, in this case, protein supplements, and a sports store as one that sells sports-related material (clothing, footwear, sports supplements, backpacks, and equipment).

### 2.2. Sample Mineralization

For the determination of the different metals in the PS, the dry incineration method was used. For this purpose, 5 g of each sample was weighed in triplicate in porcelain capsules (Statlich, Berlin, Germany) and dried in an oven (Nabertherm, Lilienthal, Germany) for 24 h at a temperature of 60–80 °C. Then, they were transferred to an oven-muffle (Nabertherm, Lilienthal, Germany), whose temperature increased by 50 °C every hour for 24 h. Once the temperature of 425 ± 15 °C was reached, it was maintained for 24 h, thus achieving incineration of the samples. The white ashes obtained were dissolved to a volume of 25 mL in 1.5% HNO_3_ (Sigma Aldrich, Taufkirchen, Germany) [63,64,65].

### 2.3. Mineral Elements Quantification

The reference method for metal detection is Inductively Coupled Plasma Optical Emission Spectrometry (ICP-OES). For this purpose, a Thermo Scientific iCAP PRO (Waltham, MA, USA) was used, and the instrumental conditions, together with the wavelengths, limits of quantification (LQ) and detection (LD), are listed in Table A1 [66,67]. For quality control of the method (Table 3), the recovery percentages (RP) were determined using several standard reference materials under reproducibility conditions.

### 2.4. Metal Exposure Assessment: Estimated Daily Intake (EDI)

Once the levels of each of the metals in the PS samples had been determined, the dietary exposures were estimated using the Estimated Daily Intake (EDI) Equation (1) considering an adult with a body weight of 70 kg b.w. and in three consumption scenarios (30, 60, and 100 g PS/day). The first scenario (30 g/day) refers to the daily intake recommendation included in most of the labeling of these PS, the second (60 g/day) refers to those individuals who consume two shakes daily, and the last (100 g/day) is considered the high consumption scenario.

EDI = Metal concentration detected (mg/g PS) × Amount of PS consumed (g/day)(1)

### 2.5. Nutritional and Toxicological Risk Characterization

For the nutritional characterization of the essential metals, the percentages of contribution to the EDIs to the reference intake values (NRI) established for the Spanish population were estimated (Table 1) [55,56] and Equation (2).

(2) $$\%\;\mathrm{of}\;\mathrm{contribution}\;\mathrm{to}\;\mathrm{the}\;\mathrm{NRI}=\frac{\mathrm{EDI}}{\mathrm{NRI}}\cdot 100$$

For the toxicological characterization of those potentially toxic metals (Al, Ni, Co, Sr, Ba, B, and V), Equation (3) was used to estimate the percentages of the contribution of the EDIs to the UL (Tolerable Upper Intake Level), TDI (Tolerable Daily Intake), and TWI (Tolerable Weekly Intake) shown in Table 2.

(3) $$\%\;\mathrm{of}\;\mathrm{contribution}\;\mathrm{to}\;\mathrm{the}\;\mathrm{reference}\;\mathrm{intake}=\frac{\mathrm{EDI}}{\mathrm{UL}/\mathrm{TDI}/\mathrm{TWI}}\cdot 100$$

In addition, for these potentially toxic elements and some of the essential elements, a risk assessment was carried out using the Targeted Hazard Quotient (THQ), which is defined as the quotient between the dose to which the consumer is exposed and the reference dose for each metal (RfD) (Table 4 and Equation (4)) [68,69,70]. Equation (5) was used to estimate the hazard index (HI) [69,71]. If the HI results ≥ 1, a moderate or high risk to the consumer’s health should be expected [72,73,74].

(4) $$\mathrm{THQ}=\frac{\mathrm{Exposure}\;\mathrm{dose}}{\mathrm{RfD}}=\frac{\frac{E_{f}\cdot C_{metal}\cdot D_{i}\cdot E_{d}}{B_{w}\cdot A_{t}}\cdot 10^{-3}}{\mathrm{RfD}}$$

- *E_f_*: exposure frequency (365 days/year).
- *C_metal_*: average concentration of each metal in PS (mg/kg).
- *D_i_*: daily intake of PS (30, 60 y 100 g/day).
- *E_d_*: average duration of exposure to PS (25 years).
- *B_w_*: average weight (70 kg b.w.).
- *A_t_*: average exposure time (*E_f_* · *E_d_*).

HI = Sum THQ(5)

### 2.6. Statistical Analysis

The statistical analysis was carried out with GraphPad Prism 8.1.1. (GraphPad, San Diego, CA, USA) for Windows to identify statistically significant differences (*p* < 0.05) in the levels of the different minerals and PTE in the whey protein supplements analyzed [66,76]. 

The Anderson—Darling, D’Angostino and Pearson, Shapiro—Wilk, and Kolmogorov—Smirnov normality tests were applied to study the distribution of the data, which do not follow a normal distribution [77]. Therefore, non-parametric tests such as Mann—Whitney were applied [78].

## 3. Results

### 3.1. Levels of Essential Elements and Potentially Toxic Elements in PS

PS is used by various consumer profiles to increase protein intake for different purposes; however, by consuming them, the individual is also exposing him/herself to different metals, some considered essential (Na, K, Ca, Mg, Mo, Mn, Cu, Fe, Zn, Cr, and Co) and others as PTE (Al, Ni, Sr, Ba, B, and V) (Table 5).

### 3.2. Consumption Scenario 1: 30 g Whey Protein Supplement/Day

Some PS manufacturers indicate in the labeling a recommended daily intake of 30 g/day, which would be represented in this first consumption scenario (Table 6 and Table 7). The elements found in higher proportions are Na, K, Mg, and Ca, but none of them exceeds the NRI established by AESAN.

None of the metals studied from a nutritional point of view exceeds the NRI established if the average metal concentrations determined are considered. The results indicate that some of the essential elements studied (Na, K, Mg, and Ca) are provided in high quantities by the PS, even though they contribute less than 10% of the NRI, considering the average concentration. Considering the maximum concentrations of Mg and Ca, about 30% of the NRI would be provided by a 30 g/day dose.

Mo is an essential element involved in the processing of proteins and DNA; therefore, it is necessary to provide it in adequate amounts [34,35]. The consumption of PS in these amounts (30 g/day) could provide 26.99% of the amount of the daily recommendations of Mo if the average concentration of Mo among all analyzed samples was considered. Nevertheless, as happens with all elements studied, Mo is also provided by other food sources such as beans, dairy products, leafy vegetables, cereals, and rice, the latter being widely consumed among athletes [38]. In the case of Mo, it would be easy to face situations where the consumer exceeds the NRI of 65 µg/day established [19]. If the maximum amount of Mo determined (4.26 mg/kg of PS) was considered, the estimated EDI would be almost twice the NRI (128 µg/day) and this daily exposure has been associated with liver, kidney, and reproductive system alterations [80]. 

A similar situation occurs for Cr without reaching such high contribution percentages as Mo does. Considering the average Cr concentration in PS, the consumption of 30 g PS/day contributes to almost 20% of the NRI in men (18.30%) and 25.63% in women. If the maximum concentration determined was considered, these contributions would rise to 51.17% and 71.64%, respectively. Like the rest of the metals, Cr presents several dietary sources, and it may be likely that Cr’s daily dietary intake overpasses the NRI, especially in women, because their requirements are lower. 

For all the PTE listed in Table 7 (Al, B, V, Ni, Co, Sr, and Ba), in a 30 g PS/day consumption scenario, the estimated EDIs using the average concentrations determined would represent a percentage contribution of less than 5% of the established reference values (TWI, TDI, UL). Therefore, for these PTE, the daily intake of 30 g of PS would not pose any health risk.

### 3.3. Consmption Scenario 2: 60 g Whey Protein Supplement/Day

In a consumption scenario of 60 g/day, the estimated daily intakes (EDIs), considering average concentration and the contribution percentages to the reference values, are doubled with respect to the previous scenario. Thus, the exposure to Mo and Cr in women would exceed 50% of the NRI. If the EDIs are calculated using the maximum concentrations, the consumption of 60 g/day of PS would be providing more Cr and Fe than necessary, the latter only in the case of men and women over 60 years of age (Table 6). 

The PTE B and V must be consumed daily in adequate amounts, and a daily intake of 60 g of whey PS would only provide 0.45% and 0.14% of the UL, respectively (Table 7). Considering these levels, it would be unlikely to observe alterations at the level of the development and reproductive system due to this B dietary exposure [52]. 

### 3.4. Consmption Scenario 3: 100 g Whey Protein Supplement/Day

In a high consumption scenario of PS (100 g/day) (Table 6 and Table 7), there are significant dietary exposures to the essential elements. Although K was identified as the element with the highest concentrations in these whey protein supplements, a 100 g/day consumption of these products would generate a low contribution to its NRI because of the high daily requirements set for this element (3500 mg/day). High intakes of Ca have been estimated, as predicted, since the analyzed PS are formulated from whey [81]. The EDI of Ca calculated using the maximum Ca concentration determined is more than 100% of the daily nutritional requirements, and this excess of Ca intake could be associated with health risks such as nausea, vomiting, calcification of soft tissues, fatigue, and arrhythmias [26]. 

Special attention must be given to the Na content (3382.41 mg/kg) of these whey protein supplements as this essential element is closely related to blood pressure, and excessive exposure may allow the development of arterial hypertension [21,82]. The consumer profile should be considered, as consumers with hypertension should reduce their daily exposure to this element since consumption of 30 g/day provides 101.472 mg (6.76% NRI). If the labeling of these products showed the Na content, consumers following a low Na diet could choose those products with lower Na content. 

Mg in women has a lower NRI than in men, as shown in Table 6. The EDIs of Mg in a 100 g PS/day consumption scenario would show higher percentages of contribution to the NRI in women, reaching 100% if the maximum concentrations determined are considered. As Mg has several dietary sources, high consumers of whey PS may be at risk of overpassing the NRI. Therefore, these Mg-rich whey PS would not be a good option for women and probably not for men either. Including the Mg levels in the nutritional information label would contribute to the management and communication of this risk. Consumers should be informed of this content.

In the case of Zn, if the EDIs for men and women are calculated using the maximum concentrations of Zn detected, men would be exposed to almost 100% of the NRI, and women would exceed their NRI just by the consumption of 100 g/day of whey PS. 

Table 7 shows the toxicological evaluation of those potentially toxic elements (PTE) and shows that none of the EDIs presents a percentage contribution to the reference values (UL, TDI, and TWI) above 10%. This risk characterization shows that the amount of Ni, Co, Sr, Ba, B, V, and Al contributed by the daily intake of 100 g of PS does not pose a toxic risk due to the occurrence and exposure to these elements. 

Considering the average levels of Al determined in the PS, the percentages of contribution to the Al TWI are for all three consumption scenarios below 10%. Moreover, exposure to this PTE through whey protein supplements is not identified as a risk to health in this study.

### 3.5. Targeted Hazard Quotient (THQ)

Considering the average levels of the metals for which the US EPA has established an RfD, the calculation of THQ and HI is shown in Table 8 for three consumption scenarios. It has been considered that the whey PS has been consumed daily for over 25 years.

According to the results shown in Table 8, the daily intake of 30, 60, and 100 g of whey PS for 25 years does not pose a health risk since the hazard index (HI) is less than one in these consumption scenarios. The first scenario represents, as indicated by some of the labels of the PS analyzed, one serving. This amount is commonly ingested by gym users either in their pre- or post-workout shakes. For the estimated HI to be greater than one, the individual must consume more than 100 g of PS daily for 25 years, exactly around 115 g daily.

The essential element to which the consumer is exposed in the greatest quantities is Fe. The minerals that contribute the most to HI are those with the highest THQ values, and they are Co, followed by Mo and Cr. However, if the risk characterization is carried out considering the punctual exposure to these new products, the essential elements that present the highest contribution percentages are Mo, followed by Cr and Ca. 

For the PTE, the highest contribution percentages were estimated for Al, followed by Ni and Sr, being lower than 5%. In the calculation of HI, the PTE with the highest THQ value and, therefore, the highest contribution to the calculation of HI is Ni, followed by Ba. 

Although the characterization of the risk resulting from chronic exposure to PS could not be carried out considering all the metals studied. In view of the results, it can be affirmed that those elements that present a higher THQ value are not the same as those that present a higher percentage contribution to their reference value.

## 4. Conclusions

PS are new products on the market that are consumed with the aim of increasing daily protein intake. However, among their components, there are other nutrients, such as essential elements but also some potentially toxic elements in quantities that may pose a risk to the consumer. Key outcomes of this study are the following: Mo content of PS considered showed the highest contribution percentage to the recommended intake by the authorities. The same considerations can be made for Cr with a high contribution to recommended intake for women. If we consider the amount of both metals provided not only by the PS but also by the rest of the foods, which compose the diet, it is likely that the maximum recommended amounts of Mo and Cr would be exceeded, with a potential health risk for consumers. In the case of potentially toxic elements, these situations will hardly occur as the content in PS is lower. In any case, as exposure to mineral elements affects food quality and the safety of consumers, its content should be warned on the label. This proposed risk management action could contribute not only to risk communication and consumer education but to the prevention of health risks associated with high chronic minerals consumption scenarios. In addition, promoting a European framework that regulates the maximum levels of these elements in these novel foods would contribute to ensuring consumer safety. In view of these results, it can be affirmed that PS is a source of different metals, some of them present in high amounts; thus, it is necessary to educate the population to be aware of this and that the intake of high amounts may generate health risks.

## Figures and Tables

**Table 1:** Nutritional reference intake of minerals for the adult population. Based on [55,56].

Nutrient	Gender and Age	Nutritional Reference Intake (NRI)	
Na	♂/♀	1500	mg/day
K	♂/♀	3500	mg/day
Mg	♂	350	mg/day
	♀	300	mg/day
Ca	♂/♀	950	mg/day
Mo	♂/♀	65	μg/day
Mn	♂/♀	3	mg/day
Cu	♂	1.3	mg/day
	♀	1.1	mg/day
Fe	♂	9.1	mg/day
	♀ 20–59 years	18	mg/day
	♀ ≥ 60 years	9	mg/day
Zn	♂	11	mg/day
	♀	8	mg/day
Cr	♂	35	µg/day
	♀	25	µg/day

NRI: the amount of nutrients required for proper functioning of the body in a given population [55,56].

**Table 2:** Toxicological reference intake values established for the potentially toxic elements (PTE) in the adult population. Based on [57,58].

Metal	Gender	Reference Value
	Tolerable Weekly Intake (TWI)	
Al	♂/♀	1 mg/kg b.w./week [59]
	Tolerable Daily Intake (TDI)	
Ni	♂/♀	13 µg/kg b.w./day [50]
Co	♂/♀	0.0016 mg/kg b.w./day [40]
Sr	♂/♀	0.13 mg/kg b.w./day [60]
Ba	♂/♀	0.2 mg/kg b.w./day [46]
	Tolerable Upper Intake Level (UL)	
B	♂/♀	0.16 mg/kg b.w./day [61]
V	♂/♀	0.026 mg/kg b.w./day [62]

TWI: maximum amount of a substance that can be consumed weekly over a lifetime without posing a risk to an individual [58]. TDI: maximum amount of substance that can be ingested daily for a lifetime without posing a risk to the health of the consumer [58]. UL: maximum amount of substance that can be consumed during a lifetime on a daily basis without any adverse effect occurring [58].

**Table 3:** Recovery study using standard reference materials (SRM).

Metal	Certified Material	Concentration Recorded (mg/kg)	Concentration Cerified (mg/kg)	Recovery Percentage (%)
Al	SRM 1515 Apple Leaves	286 ± 9	285.1 ± 26	99.7
Sr		25.0 ± 2.0	24.6 ± 4.0	98.3
Cr		0.29 ± 0.03	0.30 ± 0.00	97.8
Co		0.09 ± 0.00	0.09 ± 0.00	100
Mo		0.09 ± 0.01	0.09 ± 0.02	99.4
B		27.0 ± 2.0	27.0 ± 1.5	99.9
Na	SRM 1548a Typical Diet	8132 ± 942	8001.9 ± 4.76	98.4
Ca		1967 ± 113	161.1 ± 158	99.7
K		6970 ± 125	6858.5 ± 318	98.4
Mg		580 ± 26.7	575 ± 25.7	98.1
Ni		0.37 ± 0.02	0.38 ± 0.04	102.3
Ba		1.10 ± 0.10	1.13 ± 0.09	102.5
Zn	SRM 1567a Wheat Flour	11.6 ± 0.4	11.4 ± 0.2	98.2
Mn		9.4 ± 0.9	9.3 ± 0.5	98.9
Fe		14.1 ± 0.5	13.9 ± 0.3	98.9
V		0.011 ± 0.00	0.011 ± 0.00	99.4
Cu		2.1 ± 0.2	2.09 ± 0.4	99.7

**Table 4:** The reference doses (RfD) for metals. Based on [75].

Metal	Reference Dose (RfD)
Mn	0.14 mg/kg/day
Cu	4 × 10^−2^ mg/kg/day
Zn	0.3 mg/kg/day
Fe	0.7 mg/kg/day
Cr	3 × 10^−3^ mg/kg/day
Mo	5 × 10^−3^ mg/kg/day
Ni	2 × 10^−2^ mg/kg/day
Co	3 × 10^−4^ mg/kg/day
V	5.04 × 10^−3^ mg/kg/day
Ba	0.07 mg/kg/day
Sr	0.6 mg/kg/day
Al	4 × 10^−4^ mg/kg/day
B	0.2 mg/kg/day

**Table 5:** Average, maximum and minimum concentration of essentials elements and potentially toxic elements in PS. Based on [79].

	Metal	C_average_ (mg/100 g) (C_min_–C_max_)		Metal	C_average_ (mg/100 g) (C_min_–C_max_)
Essential elements	Na	338.241 (23.978–1142.736)	Potentially Toxic Elements (PTE)	Al	0.719 (0.040–3.522)
	K	468.910 (23.907–969.435)		Ni	0.032 (0.001–0.140)
	Mg	80.959 (5.753 -324.844)		Sr	0.284 (03.7–1.036)
	Ca	381.127 (47.602–1100.050)		Ba	0.101 (0.023–0.505)
	Mo	0.058 (0.005–0.426)		B	0.084 (<LQ–1.056)
	Mn	0.302 (0.009–2.687)		V	0.004 (<LQ–0.014)
	Cu	0.257 (0.038–1.042)			
	Fe	2.574 (0.219–17.564)			
	Zn	1.460 (0.135–10.151)			
	Cr	0.021 (0.003–0.060)			
	Co	0.007 (<LQ–0.031)			

**Table 6:** Estimated Daily Intakes (EDI) and contributions to NRI of esssential elements when consuming 30, 60 and 100 g of whey PS/day.

Consumption Scenario				30 g PS/day		60 g PS/day		100 g PS/day	
Metal		C_averge_ (mg/kg) (Range)	Gender/Age	EDI_average_ (mg/day) (Range)	Average % Contribution to NRI (Range)	EDI_average_ (mg/day) (Range))	Average % Contribution to NRI (Range)	EDI_average_ (mg/day) (Range)	Average % Contribution to NRI (Range)
Essential elements	Na	3382.41 (239.78–11427.36)	♂/♀	101.472 (7.194–342.821)	6.76 (0.48–22.85)	202.945 (14.387–685.642)	13.53 (0.96–45.71)	338.241 (23.978–1142.736)	22.50 (1.60–76.18)
	K	4689.10 (239.07–9694.35)	♂/♀	140.673 (7.172–290.830)	4.02 (0.20–8.31)	281.346 (14.344–581.661)	8.04 (0.41–16.62)	468.910 (23.907–969.435)	13.50 (0.68–27.70)
	Mg	809.59 (57.53–3248.44)	♂	24.288 (1.726–97.453)	6.94 (0.49–27.84)	48.576 (3.452–194.907)	13.88 (0.99–55.69)	80.959 (5.753–324.844)	23.13 (1.64–92.81)
			♀		8.10 (0.58–32.48)		16.19 (1.15–64.97)		26.99 (1.92–108.28)
	Ca	3811.27 (476.02–11000.50)	♂/♀	114.338 (14.281–330.015)	12.04 (1.50–34.74)	228,676 (28.561–660,030)	24.07 (3.01–69.48)	381.127 (47.602–1100.050)	40.12 (5.01–115.79)
	Mo	0.58 (0.05–4.26)	♂/♀	0.018 (0.002–0.128)	26.99 (2.42–196.60)	0.035 (0.003–0.256)	53.99 (4.84–393.20)	0.058 (0.005–0.426)	89.98 (8.07–655.33)
	Mn	3.02 (0.09–26.87)	♂/♀	0.091 (0.003–0.806)	3.02 (0.09–26.87)	0.181 (0.005–1.612)	6.04 (0.17–53.73)	0.302 (0.009–2.687)	10.07 (0.29–89.56)
	Cu	2.57 (0.38–10.42)	♂	0.077 (0.011–0.312)	5.92 (0.88–24.04)	0.154 (0.023–0.625)	11.85 (1.75–48.08)	0.257 (0.038–1.042)	19.75 (2.92–80.13)
			♀		7.00 (1.04–28.41)		14.00 (2.07–56.82)		23.34 (3.45–94.70)
	Fe	25.74 (2.19–175.64)	♂	0.772 (0.066–5.269)	8.49 (0.72–57.90)	1.544 (0.131–10.538)	16.97 (1.44–115.80)	2.574 (0.219–17.564)	28.28 (2.40–193.01)
			♀ 29–59 years		4.29 (0.36–29.27)		8.58 (0.73–58.55)		14.30 (1.21–97.58)
			♀ ≥ 60 years		8.58 (0.73–58.55)		17.16 (1.46–117.07)		28.60 (2.43–195.15)
	Zn	14.60 (1.35–101.51)	♂	0.438 (0.041–3.045)	3.98 (0.37–27.69)	0.876 (0.081–6.091)	7.96 (0.74–55.37)	1.460 (0.135–10.151)	13.27 (1.23–92.29)
			♀		5.47 (0.51–38.07)		10.95 (1.02–76.14)		18.25 (1.69–126.89)
	Cr	0.21 (0.03–0.60)	♂	0.006 (0.001–0.018)	18.30 (2.35–51.17)	0.013 (0.002–0.036)	36.61 (4.71–102.34)	0.021 (0.003–0. 060)	61.01 (7.85–170.57)
			♀		25.63 (3.30–71.64)		51.25 (6.59–143.28)		85.42 (10.99–238.80)
	Metal	C_averge_ (mg/kg) (C_min_–C_max_)	Gender	EDI_average_ (mg/day) (EDI_min_–EDI_max_)	% contribution to TDI	EDI_average_ (mg/day) (EDI_min_–EDI_max_)	% contribution to TDI	EDI_average_ (mg/day) (EDI_min_–EDI_max_)	% contribution to the TDI
	Co	0.07 (<LQ–0.31)	♂/♀	0.002 (X–0.009)	1.74 (X–8.43)	0.004 (X–0.019)	3.49 (X–16.86)	0.007 (X–0.031)	5.82 (X–28.10)

NRI: the amount of nutrients required for proper functioning of the body in a given population [55,56]. TDI: maximum amount of substance that can be ingested daily for a lifetime without posing a risk to the health of the consumer [58].

**Table 7:** Estimated Daily Intakes (EDI) and contributions to reference intake values of PTE when consuming 30, 60 and 100 g of whey PS/day.

	Consumption Scenario			30 g PS/Day		60 g PS/Day		100 g PS/Day	
Potentially Toxic Elements (PTE)	**Metal**	**C_averge_ (mg/kg)** **(Range)**	**Gender**	**EDI_average_ (mg/Day)** **(Range)**	**Average % Contribution to TWI** **(Range)**	**EDI_average_ (mg/day)** **(Range)**	**Average % Contribution to TWI** **(Range)**	**EDI_average_ (mg/day)** **(Range)**	**Average % Contribution to TWI** **(Range)**
	Al	7.19 (0.40–35.22)	♂/♀	0.216 (0.012–1.057)	2.16 (0.12–10.57)	0.431 (0.024–2.113)	4.31 (0.24–21.13)	0.719 (0. 040–3.522)	7.19 (0.40–35.22)
	Metal	C_averge_ (mg/kg) (C_min_–C_max_)	Gender	EDI_average_ (mg/day) (EDI_min_–EDI_max_)	% contribution to TDI	EDI_average_ (mg/day) (EDI_min_–EDI_max_)	% contribution to TDI	EDI_average_ (mg/day) (EDI_min_–EDI_max_)	% contribution to the TDI
	Ni	0.32 (0.01–1.40)	♂/♀	0.010 (0.0004–0.042)	1.05 (0.05–4.62)	0.019 (0.001–0.084)	2.10 (0.10–9.23)	0.032 (0.001–0.140)	3.50 (0.16–15.39)
	Sr	2.84 (0.37–10.36)	♂/♀	0.085 (0.011–0.311)	0.94 (0.12–3.42)	0.171 (0.022–0.622)	1.87 (0.25–6.83)	0.284 (0.037–1.036)	3.12 (0.41–11.38)
	Ba	1.01 (0.23–5.05)	♂/♀	0.030 (0.007–0.151)	0.22 (0.05–1.08)	0.061 (0.014–0.303)	0.44 (0.10–2.16)	0.101 (0.023–0.505)	0.72 (0.26–3.60)
	Metal	C_averge_ (mg/kg) (C_min_–C_max_)	Gender	EDI_average_ (mg/day) (EDI_min_–EDI_max_)	% contribution to UL	EDI_average_ (mg/day) (EDI_min_–EDI_max_)	% contribution to UL	EDI_average_ (mg/day) (EDI_min_–EDI_max_)	% contribution to UL
	B	0.84 (<LQ–10.56)	♂/♀	0.025 (X–0.317)	0.23 (X–2.83)	0.051 (X–0.634)	0.45 (X–5.66)	0.084 (X–1.056)	0.75 (X–9.43)
	V	0.04 (<LQ–0.14)	♂/♀	0.001 (X–0.004)	0.07 (X–0.23)	0.003 (X–0.008)	0.14 (X–0.46)	0.004 (X–0.014)	0.24 (X–0.76)

X: value not available because it is below the limit of quantification (LQ). TWI: maximum amount of a substance that can be consumed weekly over a lifetime without posing a risk to an individual [58]. TDI: maximum amount of substance that can be ingested daily for a lifetime without posing a risk to the health of the consumer [58]. UL: maximum amount of substance that can be consumed during a lifetime on a daily basis without any adverse effect occurring [58].

**Table 8:** Exposure dose, Targeted Hazard Quotient (THQ) and the Hazard Index (HI).

Metals	Daily Intake					
	30 g/Day		60 g/Day		100 g/Day	
	Exposure dose (mg/kg/day)	THQ	Exposure dose (mg/kg/day)	THQ	Exposure dose (mg/kg/day)	THQ
Mn	0.00130	0.00925	0.00259	0.01850	0.00432	0.03084
Cu	0.00110	0.02751	0.00220	0.05501	0.00367	0.09169
Zn	0.00626	0.02086	0.01251	0.04171	0.02086	0.06952
Fe	0.01103	0.01576	0.02206	0.03152	0.03677	0.05253
Cr	0.00009	0.03051	0.00018	0.06101	0.00031	0.10169
Mo	0.00025	0.05013	0.00050	0.10026	0.00084	0.16710
Ni	0.00014	0.00683	0.00027	0.01366	0.00046	0.02277
Co	0.00003	0.09305	0.00006	0.18611	0.00009	0.31018
B	0.00036	0.00181	0.00072	0.00361	0.00120	0.00602
V	0.00002	0.00374	0.00004	0.00748	0.00006	0.01247
Sr	0.00122	0.00203	0.00244	0.00406	0.00406	0.00677
Ba	0.00043	0.00621	0.00087	0.01241	0.00145	0.02069
HI = ∑ THQ		0.26768		0.53536		0.89227

## Data Availability

Data is contained within the article.

## Appendix A

**Table A1:** Inductively Coupled Plasma Optical Emission Spectrometry (ICP-OES) operating parameters.

	Inductively Coupled Plasma Optical Emission Spectrometry (ICP-OES) operating parameters		
RF power	1150 W		
Nebulizer gas flow	12.5 L/min		
Cool gas flow	12.5 L/min		
Nebulizer gas pressure	0.2 L/min		
Auxiliary gas flow	0.5 L/min		
Pump speed	45 rpm		
Metals	Emission waveleghts (nm)	Detection limits (mg/L)	Quantification limits (mg/L)
Al	167.0	0.005	0.015
B	249.6	0.008	0.027
Ba	455.4	0.0006	0.002
Ca	315.8	1.629	5.432
Co	228.6	0.001	0.005
Cr	267.7	0.001	0.005
Cu	324.7	0.003	0.011
Fe	238.2	0.004	0.013
K	766.4	1.764	5.883
Li	670.7	0.013	0.031
Mg	383.8	1.580	5.268
Mn	257.6	0.0008	0.003
Mo	202.0	0.0016	0.005
Na	818.3	2.221	7.404
Ni	221.6	0.0009	0.003
Sr	407.7	0.003	0.011
V	292.4	0.0014	0.004
Zn	213.8	0.0027	0.009
